# New insights into structure and function of bis-phosphinic acid derivatives and implications for CFTR modulation

**DOI:** 10.1038/s41598-021-83240-x

**Published:** 2021-03-25

**Authors:** Sara Bitam, Ahmad Elbahnsi, Geordie Creste, Iwona Pranke, Benoit Chevalier, Farouk Berhal, Brice Hoffmann, Nathalie Servel, Nesrine Baatalah, Danielle Tondelier, Aurelie Hatton, Christelle Moquereau, Mélanie Faria Da Cunha, Alexandra Pastor, Agathe Lepissier, Alexandre Hinzpeter, Jean-Paul Mornon, Guillaume Prestat, Aleksander Edelman, Isabelle Callebaut, Christine Gravier-Pelletier, Isabelle Sermet-Gaudelus

**Affiliations:** 1 INSERM U1151, Institut Necker Enfants Malades, Université de Paris, 75015 Paris, France; 2 Muséum National d’Histoire Naturelle, UMR CNRS 7590, Institut de Minéralogie, de Physique des Matériaux et de Cosmochimie, Sorbonne Université, 75005 Paris, France; 3 UMR 8601 CNRS, Laboratoire de Chimie et Biochimie Pharmacologiques et Toxicologiques (LCBPT), Université de Paris, 75006 Paris, France; 4 Centre de Référence Maladies Rares Mucoviscidose et Maladies du CFTR, European Reference Network for Rare Respiratory Diseases, Hôpital Necker Enfants Malades, 75015 Paris, France

**Keywords:** Cell biology, Diseases, Medical research, Molecular medicine

## Abstract

C407 is a compound that corrects the Cystic Fibrosis Transmembrane Conductance Regulator (CFTR) protein carrying the p.Phe508del (F508del) mutation. We investigated the corrector effect of c407 and its derivatives on F508del-CFTR protein. Molecular docking and dynamics simulations combined with site-directed mutagenesis suggested that c407 stabilizes the F508del-Nucleotide Binding Domain 1 (NBD1) during the co-translational folding process by occupying the position of the p.Phe1068 side chain located at the fourth intracellular loop (ICL4). After CFTR domains assembly, c407 occupies the position of the missing p.Phe508 side chain. C407 alone or in combination with the F508del-CFTR corrector VX-809, increased CFTR activity in cell lines but not in primary respiratory cells carrying the F508del mutation. A structure-based approach resulted in the synthesis of an extended c407 analog G1, designed to improve the interaction with ICL4. G1 significantly increased CFTR activity and response to VX-809 in primary nasal cells of F508del homozygous patients. Our data demonstrate that in-silico optimized c407 derivative G1 acts by a mechanism different from the reference VX-809 corrector and provide insights into its possible molecular mode of action. These results pave the way for novel strategies aiming to optimize the flawed ICL4–NBD1 interface.

## Introduction

Cystic fibrosis (CF) is a life limiting autosomic recessive genetic disease whose most frequent mutation is the deletion of phenylalanine 508 (p.Phe508del, F508del thereafter) in the first nucleotide-binding domain (NBD1) of the Cystic Fibrosis Transmembrane Conductance Regulator protein (CFTR)^1^. CFTR is a cAMP-dependent chloride (Cl^−^) channel encoded by the *CFTR* gene^2^. CFTR encompasses two Membrane Spanning Domains (MSDs) that form an anion selective pore, two Nucleotide Binding Domains (NBD1 and NBD2), which contain ATP-binding sites and a regulatory region (R)^3^. The F508del mutation affects the thermodynamic stability of NBD1 but also its interfaces with the MSDs and NBD2, which compromises their assembly^4–7^. This results in inefficient folding of F508del-CFTR and its degradation^8–10^. The consequence is a dysregulation of the epithelial fluid transport in the airway epithelium, and the production of a thickened mucus favoring chronic bacterial colonization with sustained inflammation and ultimately respiratory failure^11^.

Proof of concept studies with the VX-770 CFTR potentiator demonstrated that restoring the functional defects of gating mutations, such as the p.Gly551Asp-CFTR, was associated with amazing clinical benefits^12^. Its combination with the trafficking corrector VX-809, which increases F508del-CFTR plasma membrane delivery, improved CFTR function in primary epithelial respiratory cells bearing the F508del mutation^13^. Recent data from next generation correctors highlight the necessity to combine several correctors for full restoration of F508del-CFTR function^14–16^. However, these therapies result in an improvement of respiratory function reaching a ceiling effect of ~ 15%, highlighting that there is still an unmet need to identify more potent correctors of F508-del CFTR folding defects (15).

In previous studies, we identified several compounds potentially correcting F508del-NBD1. The most active was a small chemical compound referred to as ethane-1,2-diylbis(phenylphosphinic acid) (c407) (PubChem CID: 348,520; https://pubchem.ncbi.nlm.nih.gov/compound/348520)^17,18^.

Aiming to gain further insights into the mechanism of action of this corrector and optimize its efficacy, we performed a 3 step study (i) we mapped the putative binding site on the 3D structures of both isolated NBD1 and the whole MSDs:NBDs assembly; (ii) we designed and synthetized novel derivatives to improve correction efficacy; (iii) we investigated the effect of the combination of the most potent derivative with VX-809.

Here we report the activity of a c407 derivative, designed in silico, which individually exhibited limited rescue of F508-CFTR, but in combination with VX-809 increased functional restoration of CFTR in airway cells. This molecule could be used as a molecular template to design molecules with improved efficacy to optimize the flawed ICL4–NBD1 interface.

## Results

### c407 displays a corrector activity

**Figure 1:**
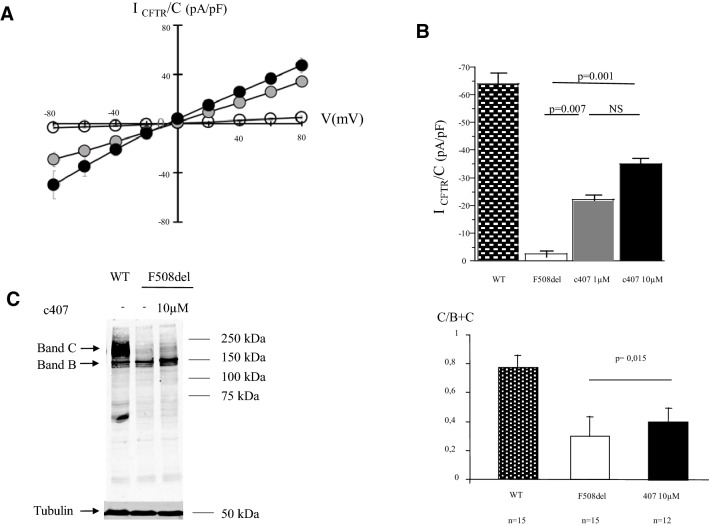
c407 activity in HeLa cells and Human nasal primary cells. (**A**) Whole cell patch-clamp experiments. Mean (SEM) CFTR-related current/voltage relationships obtained in HeLa cells stably expressing F508del-CFTR after 48 h incubation with NaCl 0.9% (empty circle, n = 5); c407 1 µM (grey circle, n = 14) and 10 µM (black circle, n = 9). Current densities normalized to cell capacitance (I_CFTR_/C) were calculated as the differences between current values in the presence of CPT-cAMP 400 µM/IBMX 100 µM minus current values after inhibition with CFTRinh-172 5 µM. (**B**) Summary of the mean CFTR current amplitudes recorded at − 60 mV and normalized to cell capacitance of the experiments in A. Results are also shown for Wild Type (WT) CFTR cells (black dotted; n = 10). Wilcoxon-test statistics are shown. (**C**) Immunoblot of HeLa cells stably expressing F508del-CFTR after incubation with c407 at 10 µM for 48 h, and NaCl 0.9% as negative control. Arrows indicate CFTR immature core glycosylated band B and complex glycosylated mature band C. CFTR was immunoblotted with monoclonal CFTR antibody 660, at 1:1000. Representative experiments of n = 12. (**D**) Summary of CFTR expression quantification. CFTR expression is evaluated by the ratio C/B + C. The ratio is significantly higher in F508del-HeLa cells incubated with c407 at 10 µM (n = 12 different experiments) *versus* F508del-HeLa cells incubated with control water (n = 15); *p* = 0.015 (Wilcoxon non parametric test).

C407 displayed a dose-dependent corrector efficacy from 1 to 10 µM in HeLa cells stably expressing F508del CFTR. This is shown by measuring I_CFTR/cAMP_ in whole cell patch clamp experiments (Fig. 1A,B). Cells treated with 1 µM c407 displayed a significant improvement in I_CFTR/cAMP_ at − 60 mV (*p* = 0.007). The 10 µM concentration increased the compound’s efficacy in comparison to 1 µM and recovered up to 54% (8) of WT-CFTR activity (*p* = 0.001). This functional restoration was supported by the increased expression of F508del-CFTR in immunoblot analysis (Fig. 1C).

In F508del homozygous human primary respiratory cells, c407 activity was inconsistent (Supplementary Fig. 1). c407 did not significantly change the CFTR dependent Cl^−^ transport at 10 µM or 50 µM concentrations, as assessed by the response to cAMP agonist Forskolin and M3-isobutyl-1-methylxanthine recorded by Short-circuit current (∆Isc/Forsk), in contrast to VX-809. Interestingly however, at the 50 µM concentration, c407 significantly improved CFTR activity in 3 out of the 11 experiments to 6.5%(0.6) (*p* = 0.05).

### Prediction of the c407 binding site

**Figure 2:**
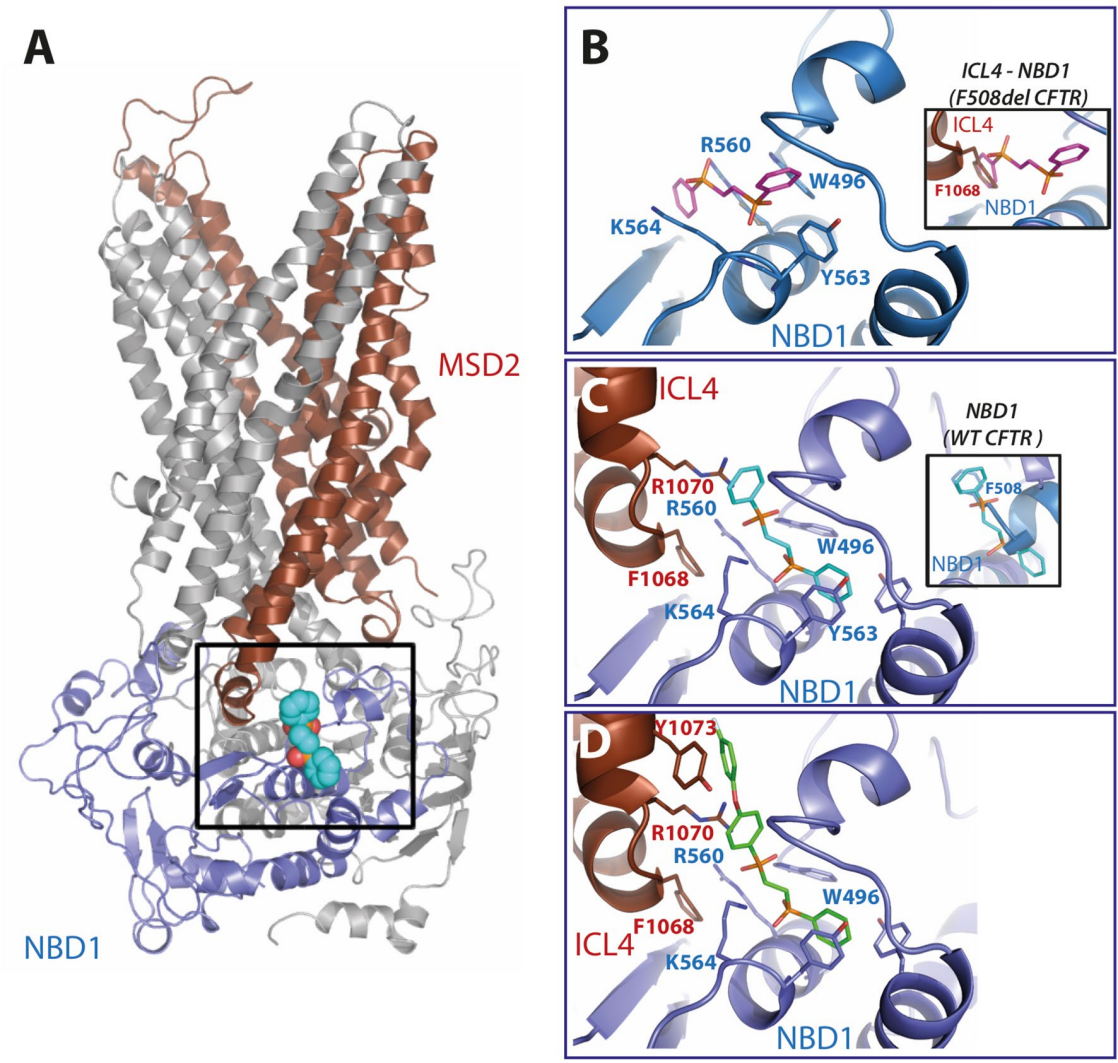
c407 interaction with the 3D structure of the F508del-CFTR MSDs:NBDs assembly. (**A**) Ribbon representation of the 3D structure model of the F508del-CFTR MSDs:NBDs assembly. NBD1 is colored in blue, MSD2 in brown red. The box highlights the position on this assembly of the putative c407 binding site, which is detailed in the right part of the figure. (**B**) Predicted c407 binding site in the context of the isolated F508del-NBD1 (extracted from the whole MSDs:NBDs architecture). In the inset is represented superimposition with the 3D structure of F508del MSDs:NBDs assembly, in order to highlight the similar position of the c407 (pink) external phenyl ring and ICL4 F1068 (red brown). (**C**) Predicted c407 binding site in the context of the F508del MSD:NBD assembly (same orientation as in Panel B). In the inset is represented the superimposition with the 3D structure of WT NBD1, in order to highlight the similar position of the c407 (green) external phenyl ring and F508 (blue). (**D**) Extended c407 analog, G1, is shown with the extension designed to interact with Y1073, thereby adding contacts with ICL4.

To identify its possible binding site, c407 was docked within the region of interest, on a F508del-CFTR 3D structure model (Fig. 2A, Supplementary Fig. 2)^19,20^. We considered NBD1 alone (Fig. 2B) or NBD1 in the MSDs:NBDs assembly (Fig. 2C). This analysis showed a common c407-binding site on NBD1, involving W496, R560, Y563 and K564 (Fig. 2B,C), similar to that published previously^17^. This allowed us to state that c407 interacts with the two basic amino acids, R560 and K564, via the negatively charged oxygen atoms of its phosphinate moieties and with Y563 and W496 by π–π stacking of its aromatic rings (Fig. 2B,C and Supplementary Fig. 3, left).

**Figure 3:**
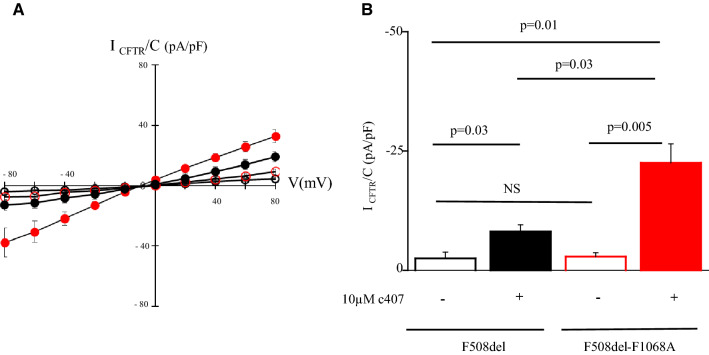
c407 restores F1068A-F508del-CFTR activity in HEK cells. (**A**) Whole cell patch-clamp experiments. Mean (SEM) CFTR-related current/voltage relationships obtained in HEK-293 cells transiently expressing F508del- or F508del-F1068A-CFTR. Current densities normalized to cell capacitance. (I_CFTR_/C) were calculated as the differences between current values in the presence of CPT-cAMP 400 µM/IBMX 100 µM minus current values after inhibition with CFTRinh-172 5 µM. Results are shown for F508del-CFTR expressing HEK cells incubated with NaCl 0.9% (black empty; n = 4) or 10 µM c407 (black; n = 7); F508del-F1068A-CFTR expressing HEK cells incubated with NaCl 0.9% (red empty; n = 4) or 10 µM c407 (red; n = 10 ). (**B**) Summary of the mean CFTR current amplitudes recorded at − 60 mV and normalized to cell capacitance of experiments in A. Wilcoxon statistics are shown.

The c407 external phenyl group occupies two different positions whether NBD1 was considered alone (Fig. 2B) or in the context of the MSDs:NBDs assembly (Fig. 2C). Indeed, in the first situation (Fig. 2B), the c407 external phenyl group is located at the position occupied by the aromatic side chain of F1068 in the intracellular Loop 4 (ICL 4) of MSD2, as shown by the superimposition of the F508del-NBD1 3D structure with that of the F508del-MSDs:NBDs assembly (inset in Fig. 2B). In the second situation, after MSD–NBD interaction (and thus in presence of F1068), the phenyl group occupies the free volume left by the missing F508, through cation–π interactions with R1070-ICL4 (Fig. 2C). This is shown by the superimposition of the F508del-MSD:NBD assembly 3D structure with that of the WT-NBD1 (inset in Fig. 2C).

The stability of c407 in its binding site was assessed through Molecular Dynamics (MD) simulations (see contact maps in Supplementary Fig. 3). Based on these simulations, binding free energy values estimated for c407 interaction, according to Molecular mechanisms/Generalized Born Surface Area (MMGBSA) calculations, were − 19 ± 5 kcal/mol (isolated NBD1) and − 19 ± 10 kcal/mol (MSDs:NBDs assembly). This suggests that c407 may act as a surrogate of critical contacts made by ICL4 for NBD1 stabilization (thanks to F1068) and/or by NBD1 for interaction with ICL4 (thanks to F508).

Comparison of MD simulations showed a global higher flexibility of NBD1 in the “NBD1 alone” simulation (average Root Mean Square Deviation (RMSD) of 9.9 ± 1.9 Å) than in the “MSDs:NBDs assembly” simulation (average RMSD of 4.7 ± 0.8 Å). This is mainly due to more flexible loops in the “NBD1 alone” model whereas the loops are more constrained within the assembly.of MSDs with NBDs.

In the “MSDs:NBDs assembly” trajectory, contacts are maintained between c407 and W496, R560, Y563 and K564 within NBD1 and F1068 and R1070 within ICL4 (Supplementary Fig. 3, bottom right), whereas in the “NBD1” simulation model, the contact with W496 is disrupted after 30 ns, and a new contact with F494 is formed (Supplementary Fig. 3, top right).

To gain further insight into the mechanisms of action of c407, we investigated whether c407 might act by mimicking a possible stabilizing effect of the F1068-ICL4 side chain on NBD1. We hypothesized that replacement of F1068 by alanine (F1068A), which leaves an empty space at the NBD1:ICL4 interface, should allow prolonged c407 binding at this location and increase its corrector effect. Western Blot analysis in transiently transfected HEK-293 cells revealed that CFTR maturation was not abrogated for the simple mutant F1068A (Supplementary Fig. 4). This enabled us to assess the effect of c407 on the double mutant F508del-F1068A-CFTR. Both F508del-CFTR and F508del-F1068A-CFTR showed minimal activity under control conditions. Treatment with 10 µM c407 for 48 h rescued CFTR activity in the double mutant F508del-F1068A-CFTR at higher levels than for the simple F508del-CFTR, as shown by current–voltage curves upon CPT-cAMP in patch clamp experiments in HEK-293 cells (Fig. 3A,B). Indeed, c407 significantly increased the cAMP dependant CFTR Cl^−^ currents (I_CFTR/cAMP)_ by 8.4 fold (1.6) in F508del–F1068A cells (*p* = 0.005) *versus* 2.1(0.9) in F508del cells (*p* = 0.03). This corresponded to a mean rescue of 33% of WT-CFTR activity (11–66%).

**Figure 4:**
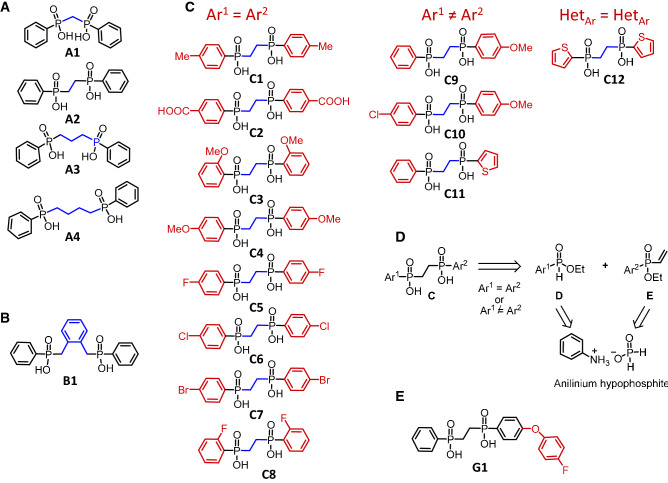
Structure of the synthesized c407 analogs. (**A**) c407 analogs with various chain length. (**B**) c407 analogs with modification of the chain rigidity. (**C**) Symmetrical or asymmetrical c407 analogs with different aromatic or heteroaromatic rings. (**D**) Retrosynthetic strategy towards compounds C1–C4, C6–C12 and G1. (**E**) Structure of the extended c407 analog, G1.

Altogether, these results supported the hypothesis that c407 may mimic, in the early steps of CFTR cooperative folding, the NBD1stabilizing effect of ICL4-F1068.

### Synthesis of c407 analogs to optimize the efficacy

As the response to c407 was inconsistent in primary cells in contrast to cell lines, we undertook the synthesis of various analogs to optimize the corrector activity of the molecule. The structure of the synthesized c407 analogs is detailed in Fig. 4A–C. The synthetic strategy is extensively described in the supplementary manuscript and Supplementary Figs. 5, 6 and 7. We first varied the length of the linker between the two phenylphosphinic groups (analogs A1–A4, Fig. 4A). Next, in order to evaluate the influence of the flexibility of this linker, the alkyl chain was replaced by a benzyl moiety resulting in analog **B1** (Fig. 4B). All these compounds were synthesized through a nucleophilic substitution of alkyl-, or benzyl-dihalides by commercially available phenyl phosphinic acids previously treated with a strong base. Finally, the synthesis of analogs **C** (Fig. 4C) displaying various aromatic groups, either identical (**C1–C8**) or different (**C9–C11**), or heteroaromatic (**C12**), was achieved. A common synthetic strategy allowing the access to both series of analogs based on a Michael addition of the deprotonated aryl phosphinic esters **D** onto aryl and vinyl ester phosphinates **E** as a key step was developed (Fig. 4D). Advantageously, both synthons D and E were synthesized from anilinium hypophosphite. Of note, compound **C5** was obtained by a similar route to compounds **A1–A4** and **B1** upon treatment of the deprotonated 4-fluoro-phenyl phosphinic acid by 1,2-dichloroethane. In order to facilitate their dissolution in aqueous medium, required for their biological evaluation, the targeted compounds were isolated as disodium salts. Based on Western Blot experiments, none of the derivatives synthesized proved more efficient than c407 (Supplementary Fig. 8).

**Figure 5:**
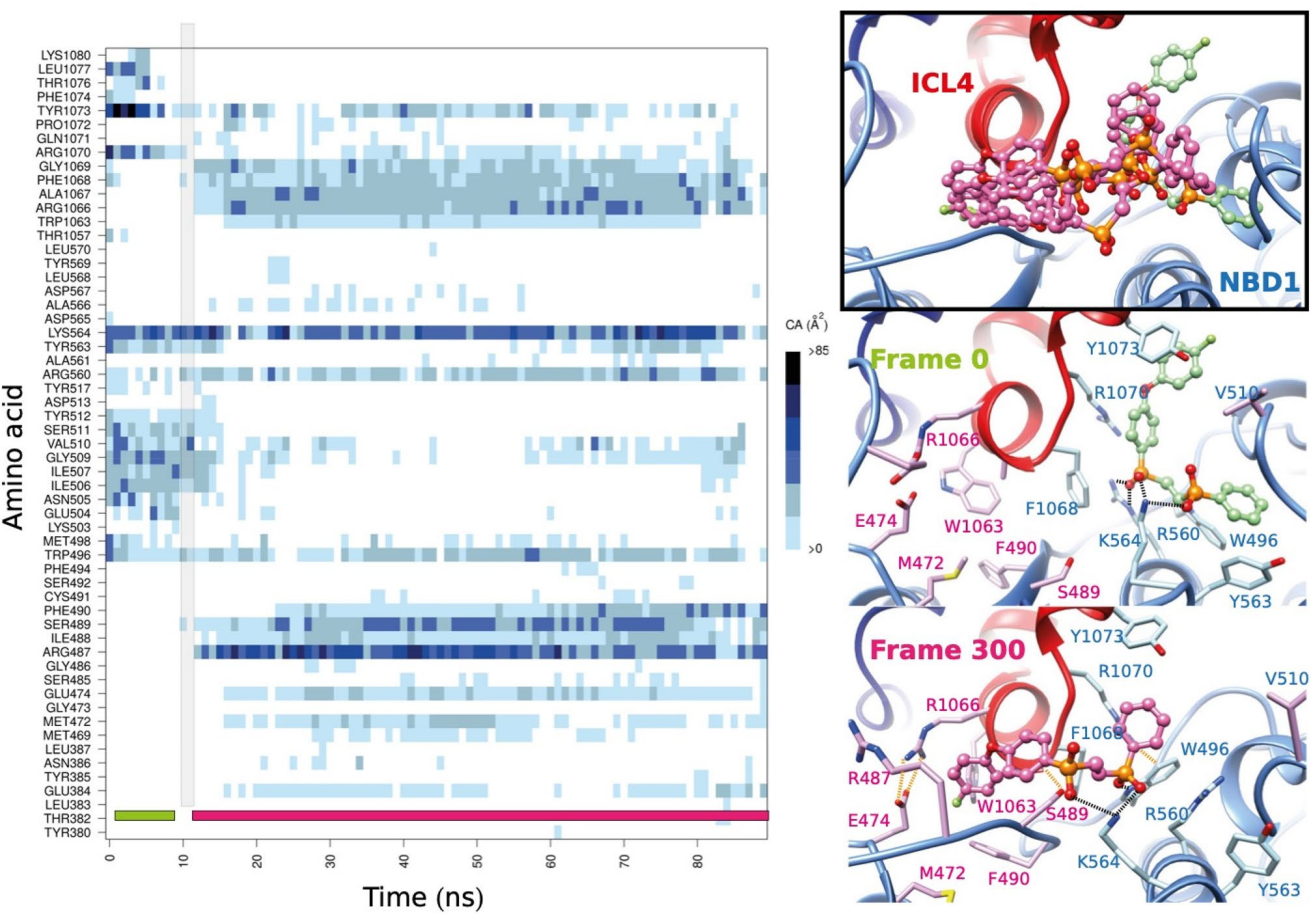
Molecular dynamics simulation of the F508del MSDs:NBDs assembly in complex with the extended c407 analog, G1. The contact map is shown on the left-hand side, with highlighted in grey, the transition area between two different positions. On the right-hand side are shown positions of G1 along the MD simulations (top), taken each 100 ns and highlighting the initial position (in green) and the position stably adopted after less then 10 ns MD simulation (pink). The middle and bottom panel illustrate the position and contacts of G1 before (Frame 0) and after 60 ns (Frame 300) MD simulation.

**Figure 6:**
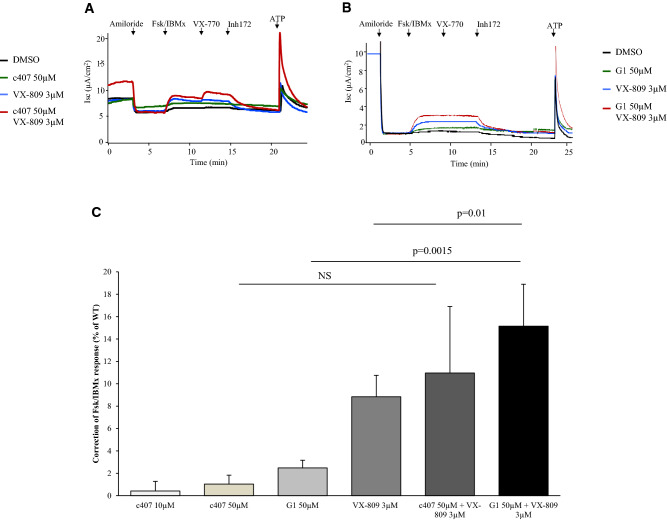
Stimulated correction of CFTR-dependent Cl^−^ secretion upon c407, its extended derivative G1, VX-809 and compounds combination. Representative Ussing chamber experiments of F508del primary nasal cells treated for 48 h with DMSO, c407 50 µM, VX-809 3 µM, or their combination (**A**) and DMSO, G1 50 µM, VX-809 3 µM, or their combination (**B**). (**C**) Shows the mean (SEM) value of the current variation in response to forskolin (10 µM)/3-isobutyl-1-methylxanthine (100 µM) and VX-770 (10 µM) in the bronchial and nasal cells of F508del homozygous patients upon incubation with c407 50 µM (n = 11), G1 50 µM (n = 14), VX-809 3 µM (n = 32), VX-809 3 µM and c407 50 µM (n = 6), and VX-809 3 µM and G1 50 µM (n = 14). Isc Forsk/VX-770 correction is calculated using the following formula: (ΔIscForsk/VX-770_corrector—ΔIscForsk/VX-770_vehicule) divided by the mean ΔIscForsk/VX-770_vehicule, and is expressed as a percentage of WT response. WT response was previously established as the current variation in response to forskolin (10 µM)/3-isobutyl-1-methylxanthine (100 µM) and VX-770 (10 µM) in the nasal cells of seven healthy control patients incubated with Ringer for 48 h. Post hoc wilcoxon statistics are shown for G1, VX-809 and their combination. The comparison between c407, VX-809 and their combination is not significant (*p* = 0.14; Kruskall Wallis test).

### Design of an extended c407 analog

As c407 was proposed to act by stabilizing and enhancing interaction at the ICL4:NBD1 interface, we hypothesized that increasing contacts at this interface would improve the activity of the small molecule. We thus designed an extended c407 molecule (analog **G1**) by adding a 4-fluoro-phenoxy substituent in the para position of one c407 phenyl group (Fig. 2D). Molecular dynamics simulations were performed on the MSDs:NBDs:**G1** complex using the same protocol as above. Unexpectedly, **G1** quickly occupied a stable, alternate position, leading to a binding free energy value of − 21 ± 9 kcal/mol (Fig. 5). The 4-fluoro-phenoxy substituent of the c407 phenyl group occupied a position roughly opposite to that adopted before MD simulation, where the other c407 phenyl group still contacted Y1073. Bonds were still established between K564 and the negatively charged oxygen atoms of the c407 phosphinate moieties. Importantly, this alternate position allowed additional contacts reinforcing the interface between ICL4 and NBD1 including contacts between (i) R1070 side chain and W496 main chain carbonyl (ii) A1067 main chain carbonyl and S489 side chain and (iii) E474 and R1066 side chains. The synthesis of the designed extended c407 analog **G1** was carried out according to a strategy similar to the one we developed for the synthesis of **C1–C11** compounds (Fig. 4E, Supplementary information and Supplementary Fig. 7).

The activity of G1 was then tested in primary HBE and HNE cells collected from 11 and 7 patients, respectively. The new compound significantly increased CFTR activity, as shown in Supplementary Fig. 1. The improvement was however very modest, reaching a mean of 4.7%(0.7) of WT-CFTR level (*p* = 0.02), as compared to VX-809 (8.5%(1.8); *p* < 0.0001).

### Combination of c407 or G1 with VX-809

The combination of c407 with VX-809 was first tested in HeLa cells. As expected, VX-809 significantly rescued F508del-CFTR activity at both 1 and 3 µM concentrations in patch clamp experiments. The combination of c407 (10 µM) with VX-809 rescued CFTR activity to 68 (6)% of WT levels for the 1 µM VX-809 concentration, and to a complete normalization to 78(17)% for the 3 µM VX-809 concentration (NS). This increase was greater than that of the addition of each compound alone (Supplementary Fig. 9). This suggests that the compounds have different mechanisms of action.

In primary cells, co-incubation with c407 50 µM and VX-809 3 µM did not significantly increase CFTR activity over that obtained with VX-809 alone (Fig. 6). In contrast, co-incubation with VX-809 3 µM and G1 50 µM significantly increased the response of VX-809, reaching a mean level of 19% (3.7) with maximal values of 45% of WT levels (*p* = 0.04).

## Discussion

Recent years have unraveled amazing progress in development of CFTR correctors. However, little mechanistic insights are available to rationalize their mode of action. We provide here possible molecular mechanisms of a CFTR corrector that we previously identified by virtual screening strategy of the 3D structure of F508del-NBD1^17^. We herein delineate a putative binding site based on an analysis combining docking/molecular dynamics simulations and site-directed mutagenesis and demonstrate that its combination with VX-809 increases CFTR activity.

The F508del mutation impairs the CFTR NBD1 domain thermodynamic stability and the cooperative NBDs-MSDs domains assembly^21,22^. The fact that “artificial” NBD1 mutations yield significant recovery of F508del channel activity demonstrates that this thermodynamic instability can be counteracted allosterically and that restoration of NBD1 thermal stability is crucial to correct F508del-CFTR folding and assembly^23–25^. Until now, molecules identified as binding NBD1, do not correct the F508del trafficking defect^26^. Classical experimental approaches have identified small molecules that correct interdomain assembly defects but fail to improve CFTR NBD1 thermostability^27^. Moreover, drugs available for CFTR correction suitable for clinical use such as VX-809, VX-661 or VX-445, only partially restore defective F508del-CFTR trafficking and have limited effects when tested individually^14,16,22^. Indeed, it has been shown that robust correction of F508del-CFTR defects requires a cooperative rescue involving distinct structural defects, including stabilization of NBD1, as well as the secondary effects of the mutation (NBD1–MSD1 or − MSD2 interfaces; NBD2 folding and NBD1–NBD2 interface)^4,6,16,28^. This was recently confirmed by clinical studies showing that a triple combination therapy based on the association of VX-770 with 2 correctors, was able to very significantly improve the respiratory status of patients in association with CFTR restoration to more than 80% of the normal^14,15^. Altogether, these results suggest that one of the challenges of F508del-CFTR protein therapy is to develop small molecules that will not only bind given subdomains of NBD1, but also form the physical connection across NBD1 and MSDs.

This led us to undertake rational design of CFTR stabilizers based on computational docking and pharmacophore building. The identified index corrector, c407, has a corrector effect in HeLa and HEK cell lines, as assessed by patch clamp experiments. Based on 3D structure data of F508del-NBD1 in the CFTR multi-domain environment, we provide data in support of a putative c407-binding site located at the NBD1:ICL4 interface level . The model of the whole MSDs:NBDs assembly we used^19^ is in agreement with the more recent cryo-EM 3D structures of human CFTR^20,29^. This is particularly true for the ICL4:NBD1 interface, specifically considered here, in which we incorporated the specific features of F508del-NBD1 observed experimentally. The role of c407 appeared critical at this NBD1:ICL4 interface, substituting for either the phenyl side chain of ICL4 F1068 (when docked on NBD1 alone) or the WT-CFTR F508 (when docked on the MSDs:NBDs assembly). The likelihood of these binding modes was further supported by molecular dynamics simulations of the protein:drug complexes. This hypothesis was also experimentally supported by site-directed mutagenesis of F1068 to an alanine, whose limited side chain leaves room for c407 binding in the “NBD1”-alone mode, even when the MSDs:NBDs assembly is achieved. Altogether, we suggest that c407 (i) mimics the phenyl side chain of F1068 during the first steps of the co-translational folding process and therefore acts as a surrogate of ICL4-F1068, transiently stabilizing F508del-NBD1 and (ii) substitutes the missing F508 side chain when MSD2 associates to form the whole MSD:NBDs assembly. This observation further supports the critical role that F1068 may play at the MSD–NBD1 interface in the global folding process. To our knowledge, this is the first study, assessing by site-directed mutagenesis the likelihood of a predicted corrector-binding site.

Interestingly, our data provide evidence for an additive effect between c407 and VX-809 in cell lines, suggesting that the compounds have different mechanisms of action. Possible binding sites for VX-809 have been reported at the interface between ICL4 and NBD1, in a region similar to the possible c407-binding site^30^. However, other studies suggest that correction of F508del involves an allosteric coupling with the NBD1:ICL4 interface, rather than direct binding^8,31,32^. We thus cannot exclude that both drugs, VX-809 and c407, act in an allosteric way on different sites.

The corrector effect of c407 could not be evidenced in primary cells, unlike in HEK and HeLa cell lines. This might be due to differences between the cell lines and the primary cells, considering the transcript level, the proteomic profile in the organelles, cytoplasm or at the membrane. Moreover, the patch clamp technique used in cell lines is more sensitive than Short Circuit Current used in primary cells in detecting changes in CFTR activity as it directly addresses CFTR activity at the cell and channel level while Ussing chamber experiments indirectly evaluate CFTR activity at the epithelial level.

The fact that in the context of primary respiratory cells, CFTR activity was significantly increased neither by c407 alone nor by the combination with VX-809, pushed us to investigate optimized compounds for greater folding efficacy. A chemistry program dedicated to modulate both the nature of the linker between the phosphorus atoms of c407 and the aromatic substituents was undertaken and resulted in the synthesis of a limited set of 16 c407 analogs with chains displaying various lengths and rigidity between phosphorus atoms or symmetrical or asymmetrical aromatic groups. However, their screening did not reveal any significant improvement of their activity as compared to c407.

To improve c407 derivatives activity, we reasoned that the most efficient path was to increase contacts between NBD1 and ICL4. The best candidate, designed in silico, displayed a 4-fluoro-phenoxy substituent in the para position of one of the c407 phenyl group. Molecular dynamics simulations indicated that this c407 derivative G1, stabilized the NBD1:ICL4 interface, involving bonds not only with the same NBD1 basic amino acids as for c407, but also between the ICL4 and the 4-fluoro-phenoxy substituent which interacts with the ICL4 coupling helix, in a position leading to a global enhancement of NBD1:ICL4 contacts. The biological activity of the compound was improved, albeit modestly, but the most striking result was the improved efficacy of its combination with VX-809 to a mean of 19% of CFTR activity in primary cells, with a maximum of 45%.

These results suggest that this increased NBD1:ICL4 interface by the c407 extended derivative G1 is biologically relevant and could therefore be used as a molecular template to design molecules with improved activity. This would lead to novel strategies for optimization of c407-derived compounds. We postulate that this observation could have important consequences for therapeutic strategies aimed at modulating the dynamics of this interface and addressing corrections of mutations affecting this interface.

## Conclusion

Our study is one of the first addressing structure-based design of drugs aiming at restoring CFTR folding. These results support the feasibility of such structure-guided research strategy framework for novel F508del correctors. This paves the way for new development based on collaborative efforts involving bioinformatics, chemistry, biology and translational medicine. Such strategy could be of value for the development of a new CFTR corrector.

## Methods

### Cell culture and media

HeLa cells, HEK-293 cells expressing human forms of CFTR or F508del-CFTR were cultured as previously described^33^.

Primary respiratory cells were obtained either from nasal brushing (HNE) or from bronchial explants (HBE) in F508del homozygote patients after written informed consent of all participants. All experiments were performed in accordance with the French Jardet law on human research. The study was approved by the Ile de France 2 Ethics Committee (Comité de Protection des Personnes Ile de France II). After achieving ~ 2 × 10^6^ cells expansion, cells were cultured in air–liquid interface with UG2% basal medium changed daily for 3–4 weeks to establish an Air Liquid Interface (ALI) culture as previously described^34^.

When the cells were 90% confluent and the transepithelial resistance > 600 Ω/cm^2^, primary HNE cell cultures were incubated at the basal side for 48 h with c407 1–50 µM diluted in NaCl 0.9%, or VX-809 1–3 µM diluted in DMSO.

### Functional assays for CFTR activity

#### Patch-clamp

Experiments were done in the whole-cell configuration in HeLa or HEK-293 cells expressing WT or F508del-CFTR following the experimental protocol described in^17^. Cells were incubated for 48 h at 37 °C with or without c407 1 or 10 µM in the assay media and VX-809 1–3 µM to test the synergy.

Current recordings were performed using the whole cell nystatin-perforated patch-clamp configuration by application of regular pulses of − 60 mV for 1 s, with a holding potential of 0 mV and an interval of 3 s. To establish the current–voltage (I–V) curves, voltage jumps were applied (1-s duration each) toward membrane potentials between − 100 and + 80 mV. CFTR Cl^−^ currents (I_CFTR_) were activated using 400 µM 8-(4-chlorophenylthio)-cAMP sodium salt (CPT-cAMP) and 100 µM 3-isobutyl-1-methylxanthine (IBMX), to achieve steady-state after 5–7 min (I_CFTR/cAMP_). Cells were then bathed with 5 µM of the specific CFTR inhibitor thiazolidinone CFTRinh-172 (Calbiochem, Germany), added to the CPT-cAMP solution to stop CFTR activation. CFTR currents were defined by the current amplitude recorded during maximum stimulation by CPT-cAMP and the inhibition with CFTRinh-172.

#### Ussing chamber recordings

The cell culture inserts were mounted into 2 Ussing hemi-chambers (VCC MC8; Physiologic Instruments) to record the trans-epithelial current, in voltage-clamp mode (0 mV) and gazed with 95% O_2_/5% CO_2_ at 37 °C, as reported in^34^. The difference between the basolateral bath solution (145 mM NaCl, 3.3 mM K_2_HPO_4_, 10 mM HEPES, 10 mM D-Glucose, 1.2 mM MgCl_2_, 1.2 mM CaCl_2_; pH 7.35), and the apical solution (where 145 mM NaCl was replaced by 145 mM Na-Gluconate) provided a basolateral to apical Cl^−^ gradient. Short-circuit current (Isc) was measured with EVC4000 Precision V/I Clamp (World Precision Instruments) and registered using PowerLab 4/30 workstation (AD Instruments, Castle Hill, Australia). During continuous recording of Isc in voltage-clamp mode, the following inhibitors and activators were added at the apical face after stabilization of baseline Isc: Na^+^ channel blocker Amiloride (100 µM) to inhibit the apical epithelial sodium channel (ENaC); cAMP agonists Forskolin (10 µM) and M3-isobutyl-1-methylxanthine (IBMx 100 µM) to activate the transepithelial cAMP-dependent current (including Cl^−^ transport through CFTR channels) (∆Isc/Forsk); VX-770 (10 µM) to potentiate CFTR activity; CFTR inhibitor Inh-172 (5 µM).

The data from HNE and HBE were pooled. This was based on a preliminary study showing robust correlation between nasal and bronchial cultures from the same subject in 6 different non CF individuals in both stimulated and inhibited CFTR activity, as well as similar levels of correction by VX-809 3 µM in bronchial and nasal primary cell cultures from F508del homozygous patients (9 HBE, 22 HNE).

### Biochemical assays for CFTR expression

Cells expressing CFTR or F508del-CFTR were incubated for 48 h at 37 °C with or without c407 10 µM or VX-809 1–3 µM in the assay media. After incubation, cells were harvested in ice-cold-(PBS) solution and pelleted at 1500 g at 4 °C. Cell pellets were lysed in RIPA buffer (150 mM NaCl, 20 mM Tris, 1% Triton X-100, 0.1% SDS, 0.5% sodium deoxycholate, pH 8.0) plus protease inhibitor mixture (1:250; Roche) for 30 min on ice. Lysates were spun for 10 min at 10,000 g at 4 °C. Western blots experiments were done as described in^33^. Briefly, 30 µg total protein was incubated at room temperature during 5 min in Laemmli buffer with 5% β-mercaptoethanol and transferred onto a 7% Tris–glycine SDS gel. The gel was electro-transferred onto nitrocellulose membranes over 2 h at 4 °C in Tris–glycine buffer (Biorad) at 200 mA.

Proteins were then immunoblotted for 1 h at room temperature with monoclonal CFTR antibody (CFTR 660, from UNC antibody distribution program, Chappel Hill, NC, USA) at 1:1000 ; α-tubulin (Santa Cruz, Heidelberg, Germany) at 1:1000.and Na–K-ATPase (ABCAM, Cambridge, UK). at 1:1000.

### CFTR 3D structures: modeling, docking and molecular dynamics simulations

#### Comparative modeling

We used a previously published model of the 3D structure of the WT-CFTR MSD:NBD assembly [amino acids 65–649 (MSD1:NBD1), including the regulatory insertion (403–435); amino acids 845–1446 (MSD2:NBD2) and the linker insertion (1182–1202)]. This model corresponds to an open form of the channel^19^ and does not include the R region and the N- and C-terminal extremities. The interface between NBD1 and ICL4, comprising Phe508, is similar to the one observed in the published cryo-EM 3D structure of WT human CFTR (pdb 6MSM) (Supplementary Fig. 2)^20^. The F508del assembly model was constructed using SwissPdbViewer^35^ by replacing coordinates of amino acids 490–530 of the WT construct by those found in the experimental 3D structure of human F508del-NBD1 (pdb 1XMJ^21^). This was followed by several cycles of energy minimizations.

#### Docking

Docking was made using GLIDE from the Small-Molecule Drug Discovery Suite developed by Schrödinger^36^. We performed the docking experiments both on the F508del MSDs:NBDs assembly and on the isolated F508del-NBD1, extracted from this last assembly. As the active geometry site of a protein complex heavily depends on conformational changes induced by the bound ligand, we performed induced fit docking, which allows some flexibility of the amino acid side chains. We retained the best solution for each docking based on the Standard Precision (SP) docking score.

#### Molecular dynamics simulations

Molecular dynamics simulations were performed starting from docking predictions and using CHARMM36 force field for the CFTR F508del-NBD1/c407 and CFTR F508del-[MSDs:NBDs]/c407 complexes^37^ . Simulations were also conducted with c407 derivative, G1. CHARMM compatible topology and parameter files for c407 and its derivative were generated using the CgenFF tool through the Ligand Reader & Modeler module of the CHARMM-GUI platform^38–41^. During these parameterization steps, each phosphate group was designed with a negative charge of 1, hence leading to a total charge of − 2 for each molecule.

MSD–NBD complexes were then embedded in a POPC (1-palmitoyl-2-oleoyl-*sn-*glycero-3-phosphocholine) bilayer membrane using the same protocol as previously described^29^ for generating and equilibrating protein-membrane simulations complexes, as well as for the MD simulations. Results of MD simulations were analyzed using VMD (Visual Molecular Dynamics)^42^. The stability of the protein throughout the simulations was assessed by considering RMSD values for backbone atoms. Binding free energies were calculated using the Molecular Mechanics/Generalized Born Surface Area (MMGBSA) approach through MMPBSA.py (from Ambertools 18) with default parameters, with igb = 2^43^. The contacts between ligands and CFTR/NBD1 were analyzed by VLDM (Voronoi Laguerre Delaunay for Macromolecules) which represents a complex (protein/ligand) by a Laguerre tessellation^44–46^. For consistency with the simulations, CHARMM36 parameters were included in VLDM^37^.

### Site-directed mutagenesis

Mutagenesis (substitution of the target amino-acid to an Alanine at position 1068) was performed on CFTR-WT cDNA cloned in pTracer-CMV (Invitrogen). For CFTR-WT F508del- and F508del/F1068A- expression, HEK-293 cells were transfected using lipofectamine 3000 (Thermo Fisher Scientific) with 1.5 µg of p-Tracer-CFTR. Site-directed mutagenesis was done using the QuickChange XL mutagenesis kit following manufacturer's instructions (Agilent, Les Ulis, France). Following primers were used for p.Phe508del : 5′-GCA CCA TTA AAG AAA ATA TCA TTG GTG TTT CCT ATG ATG AAT ATA G-3′ and 5′-CTA TAT TCA TCA TAG GAA ACA CCA ATG ATA TTT TCT TTA ATG GTG C-3′ and for p.Phe1068Ala : 5′-GGA CAC TTC GTG CCG CCG GAC GGC AGC CTT-3′ and 5′-AAG GCT GCC GTC CGG CGG CAC GAA GTG TCC-3′. The resulting plasmids were verified by gene sequencing.

### Chemical synthesis

Samples purified through column chromatography (Merck Kieselgel 60 (200–500 µm)),were used for MS and/or analytical data. Bruker Avance or Avance II was used to record ^1^H NMR (500 MHz), ^13^C NMR (126 MHz) and ^31^P (202 MHz) in the indicated solvent. Chemical shifts and coupling constants are given respectively in ppm and Hz. Reactions were constantly run under inert atmosphere (argon)^47^.

### Statistical analysis

Continuous variables are presented as mean(SEM). Between-group comparisons were evaluated by Mann–Whitney U test for qualitative variables and Wilcoxon test for quantitative variables. For multiple comparisons, we performed Kruskall Wallis test, Kolmogorov Smirnov test to test for normal distribution followed by post hoc Wilcoxon test.

For each test, the null hypothesis was rejected at *p* value less than 0.05. Data management and statistical analysis were performed using R Statistical software (version 3.2.0; R Foundation for Statistical Computing, Vienna, Austria).

## Supplementary Information

Supplementary Information.
