# Author Correction: New insights into structure and function of bis-phosphinic acid derivatives and implications for CFTR modulation

**DOI:** 10.1038/s41598-021-98301-4

**Published:** 2021-09-15

**Authors:** Sara Bitam, Ahmad Elbahnsi, Geordie Creste, Iwona Pranke, Benoit Chevalier, Farouk Berhal, Brice Hoffmann, Nathalie Servel, Nesrine Baatalah, Danielle Tondelier, Aurelie Hatton, Christelle Moquereau, Mélanie Faria Da Cunha, Alexandra Pastor, Agathe Lepissier, Alexandre Hinzpeter, Jean‑Paul Mornon, Guillaume Prestat, Aleksander Edelman, Isabelle Callebaut, Christine Gravier‑Pelletier, Isabelle Sermet‑Gaudelus

**Affiliations:** 1 INSERM U1151, Institut Necker Enfants Malades, Université de Paris, 75015 Paris, France; 2 Muséum National d’Histoire Naturelle, UMR CNRS 7590, Institut de Minéralogie, de Physique des Matériaux et de Cosmochimie, Sorbonne Université, 75005 Paris, France; 3 UMR 8601 CNRS, Laboratoire de Chimie et Biochimie Pharmacologiques et Toxicologiques (LCBPT), Université de Paris, 75006 Paris, France; 4 Centre de Référence Maladies Rares Mucoviscidose et Maladies du CFTR, European Reference Network for Rare Respiratory Diseases, Hôpital Necker Enfants Malades, 75015 Paris, France

Correction to: *Scientific Reports* 10.1038/s41598-021-83240-x, published online 25 March 2021

Nesrine Baatalah was omitted from the author list in the original version of this Article.

The Author Contributions section now reads:

“S.B.: conducted and acquired cellular biology experiments, analyzed and interpreted the data. A.E.: conducted and acquired molecular docking and dynamics simulations experiments, analyzed and interpreted the data. G.C: conducted and acquired chemistry experiments, analyzed and interpreted the data. I.P.: conducted and acquired cellular biology and electrophysiology experiments, analyzed and interpreted the data. B.C.: conducted and acquired cellular biology experiments, analyzed and interpreted the data. F.B.: conducted and acquired chemistry experiments, analyzed and interpreted the data. B.H.: conducted and acquired molecular docking and dynamics simulations experiments, analyzed and interpreted the data. N.S.: conducted and acquired patch clamp experiments, analyzed and interpreted the data. N.S. conducted and acquired Western Blott experiments, analyzed and interpreted the data. D.T.: conducted and acquired Western Blott experiments, analyzed and interpreted the data. A.H.: conducted and acquired and electrophysiology experiments, analyzed and interpreted the data. C.M.: conducted and acquired Western Blott experiments, analyzed and interpreted the data. M.F.D.C.: conducted and acquired cellular biology and electrophysiology experiments, analyzed and interpreted the data. AP: performed chemistry experiments. A.L.: conducted and acquired and electrophysiology experiments, analyzed and interpreted the data. A.H.i: participated to the design and interpretation of the experiments. J.P.M.: participated to the design of molecular docking experiments and interpretation of the experiments. G.P.: participated to the design of chemistry experiments and interpretation of the experiments. A.E.: conceived the study, analyzed and interpreted the data and participated to the writing of the manuscript. I.C.: designed the molecular docking and dynamics simulation analyzed and interpreted the data and participated to the writing of the manuscript. C.G.P.: designed the chemistry experiments analyzed and interpreted the data and participated to the writing of the manuscript. I.S.G.: conceived the study, analyzed and interpreted the data and wrote the manuscript”.

The original Article and accompanying Supplementary information file has been corrected.

