# Supplementary material for: New insights into structure and function of bis-phosphinic acid derivatives and implications for CFTR modulation
Source: Sci Rep. 2021 Mar 25;11:6842. doi: 10.1038/s41598-021-83240-x (PMC7994384; doi:10.1038/s41598-021-83240-x)
Supplement: Supplementary file 1 — Supplementary Information. [file 41598_2021_83240_MOESM1_ESM.docx]

**Supplementary Manuscript**

**New insights into structure and function of bis-phosphinic acid derivatives and implications for CFTR modulation**

Bitam Sara*, Elbahnsi Ahmad*, Creste Geordie*, Pranke Iwona**, Chevalier Benoit**, Berhal Farouk**, Hoffmann Brice, Servel Nathalie, Baatallah Nesrine, Tondelier Danielle, Hatton Aurelie, Moquereau Christelle, Faria Da Cunha Mélanie, Pastor Alexandra, Hinzpeter Alexandre, Mornon Jean Paul, Prestat Guillaume, Edelman Aleksander, Callebaut Isabelle***, Gravier-Pelletier Christine***, Sermet-Gaudelus Isabelle***

**Synthesis of compounds A1-A4 (modification of the chain length)**

The synthesis of these analogs has been envisaged by alkylation of commercially available phenyl phosphinic acid treated with a strong base with the corresponding alkyl dihalide (**Supplementary Figures 5A** and **5B**) ^1^.

A short screening (**Supplementary Table 1**) of the base and dihalide to be used (*n*BuLi or LDA) for the preparation of the reference compound **c407 (A2)** revealed that lithium diisopropyl amide, freshly generated by the addition of *n*-butyl lithium to diisopropylamine, as a base, and ethyl dichloride, as an alkylation agent, in THF for 66 h, gave the best yield (59 %) of the reference compound **A2** isolated after work-up and acidification with HCl 6N^2,3^. The synthesis of **c407** analogs (n = 0, 2, 3) was performed in the same conditions (**Supplementary Figure 5B** and **Supplementary Table 1**) to afford the corresponding bis-phosphinic diacids **A1**, **A3**, **A4** in moderate to good yields (**Supplementary Table 1**, entries 5-7). The disodium salts of these compounds were then generated to facilitate the dissolution of the compounds in aqueous medium, suitable for their biological evaluation. It was achieved by the addition of 2 equivalents of sodium hydroxide to each compound followed by freeze-drying.

**Typical alkylation procedure, example for c407 synthesis:**

In a flame dry round-bottomed flask were introduced under argon diisopropylamine (4.3 equiv, 7.4 mmol) and anhydrous THF (10 mL). The flask was then placed at -78°C and *n*-BuLi (4.2 equiv, 7.2 mmol) was added dropwise. After 10 min, the light-yellow solution was warmed to 0 °C and stirred for 30 min. A solution of phenyl *H*-phosphinic acid (2 equiv, 3.5 mmol) in anhydrous THF (8 mL) was then added dropwise, the yellow suspension was stirred for 30 min and 1,2-dichloroethane (1 equiv, 1.7 mmol) was introduced. The reaction mixture was allowed to warm to room temperature, then heated at reflux for 4 h and let overnight at room temperature. The mixture was concentrated under reduced pressure and the yellow resulting solid was partitioned between diethyl ether (20 mL) and aqueous NH_4_OH (70 mL). The aqueous phase was acidified with an aqueous 6M solution of HCl until the precipitation of a white solid occurred (33 mL). The solid was filtrated, washed with water followed by ether and dried under reduced pressure to afford the desired product (311 mg, 59 %). Its sodium salt was then generated in quantitative yield by the addition of 2 equivalents of sodium hydroxide followed by freeze-drying.

RMN ^1^H (CD_3_OD + NaOH, 500 MHz): δ 7.73-7.69 (m, 4H, H_Ar_), 7.41-7.33 (m, 6H, H_Ar_), 1.76 (bd, *J* = 6 Hz, 4H). RMN ^13^C (CD_3_OD + NaOH, 125 MHz): δ 139.9-138.5 (m, C_Ar_), 132.3, 131.0, 128.8 (CH_Ar_), 27.7-26.5 (m, CH_2_). RMN ^31^P CPD (CD_3_OD + NaOH, 202 MHz): δ 31.94. IR (cm^-1^) : 1436 (ν_P-CH2_), 1189 (ν_P=O_), 1163 (v), 1133 (v), 1041 (v_P-O_), 962 (ν_P-O_), 731 (v_P-C_). Tf (°C) : 264-266.

**Synthesis of compounds B1 (modification of the chain rigidity)**

The benzyl analog **B1** (**Supplementary Figure 5C**) was readily obtained in the previous conditions, in a moderate 42% yield after crystallization in acetic acid and its disodium salt was prepared in 100% yield.

RMN ^1^H (D_2_O + NaOH, 500 MHz): δ 7.54-7.40 (m, 10H, H_Ar_), 7.10 (2H, H_Ar_), 7.00 (2H, H_Ar_), 2.74 (d, *J* = 17 Hz, 4H, CH_2_). RMN ^13^C (D_2_O + NaOH, 125 MHz): δ 136.2 (d, J = 125 Hz, C_Ar_), 133.4 (C_Ar_), 131.1-130.9, 128.2-128.1, 125.9 (CH_Ar_), 36.9 (d, J = 90 Hz, CH_2_). RMN ^31^P CPD (D_2_O + NaOH, 202 MHz): δ 30.19. IR (cm^-1^) : 1715 (ν_P-OH_), 1439 (ν _P-CH2_), 1170 (ν_P=O_), 1131 (ν_P-Carom_), 954 (ν_P-O_), 753 (ν_P-C_). Tf (°C): 219-221.

**Synthesis of compounds C1-C4, C6-C12 (modulation of Ar or Het_Ar_ groups)**

The synthesis of **c407** analogs (**C1-C4, C6-C12**) displaying various aromatic or heteroaromatic groups, either identical or different was achieved thanks to a common strategy (**Supplementary Figure 6A**) involving the Michaël addition of the anion of aryl phosphinic esters **D** onto aryl and vinyl ester phosphinates **E**, as a key step. Both synthons can result from anilinium hypophosphite.

Accordingly, the synthesis of the corresponding aryl phosphinic acids **Dx** (**Supplementary Figure 6B** and **Supplementary Table 2**) was performed by Pd-catalyzed cross coupling reaction of anilinium hypophosphite with commercially available aryl iodides as electrophiles ^4^. The reaction was carried out in the presence of tetrakistriphenylphosphine palladium (0.2-1 mol %), triethylamine (3 equiv) as a base, in refluxing acetonitrile (**Supplementary Figure 6B** and **Supplementary Table 2**) to afford the corresponding crude phosphinic acids in moderate to excellent yields. Then, their esterification was performed in the presence of ethyl chloroformate and pyridine in refluxing dichloromethane to give the corresponding esters **Dx** isolated after flash chromatographic purification in 52 to 99% yield (**Supplementary Figure 6B** and **Supplementary Table 2**) ^5^. We next turned to the synthesis of the corresponding vinyl phosphinates **Ex** by the palladium-catalyzed reaction of vinyl bromide with the previously synthesized ethyl aryl phosphinates **Dx** ^6^. The reaction was performed by heating the phosphinates **Dx** with NEt_3_ and vinyl bromide in the presence of palladium dichlorobis(triphenylphosphine) (5 mol %), at 100 °C for 2 h in a sealed tube, to give the expected vinyl phosphinates **Ex** that were isolated after flash chromatographic purification in 37 to 93% yield (**Supplementary Figure 6B and Supplementary Table 2**).

With these building blocks in hand, the synthesis of symmetrical or asymmetrical analogs of compound **c407** (**Supplementary** **Figure 6C** and **Supplementary** **Table 3** was achieved by Michaël addition of the anion of the aryl phosphinic esters **Dx** onto the vinyl phosphinates **Ex** 7. The reaction was performed in the presence of a catalytic amount of *tert*-butoxy magnesium chloride (20 mol %), in THF, at room temperature until completion of the reaction (one to four days). The corresponding protected analogs of compound **c407**, **Fx**, were obtained after flash chromatographic purification in moderate to good yields ranging from 56 to 91%. Their acidic hydrolysis at 100 °C in HCl conc. led to the corresponding diacids **C1-C4, C6-C12** in 37 to 94% yield (**Supplementary Figure 6C** and **Supplementary Table 3**). They were then transformed into their disodium salt in 94 to 100% yield.

**Synthesis of the extended c407 analog G1**

It was performed according to the synthesis strategy presented above, involving the corresponding commercially available 4-​(4-​fluorophenoxy)​iodobenzene as a starting material (**Figure 7 supplementary**). The corresponding experimental procedure is detailed below.

*Synthesis of* ***Dj***: To a solution of anilinium phosphinate (275 mg, 1.68 mmol), under argon in acetonitrile (4.2 mL, C = 0.2 M) was added the commercially available 4-(4-fluorophenoxy)- iodobenzene (263 mg, 0.84 mmol) followed by triethylamine (350 µL, 2.52 mmol), palladium diacetate (3.76 mg mg, 0.017 mol) and 1,2-bis(diphenylphosphino)ethane as a ligand (7.35 mg, 0.018 mmol), the solution was then refluxed for 16 h. After cooling to r.t., the mixture was concentrated *in vacuo* and the resulting residue was diluted in H_2_O. The aqueous phase was extracted with diethyl ether then acidified to pH 1 with an aqueous solution of KHSO_4_ (1M, saturated with NaCl) and extracted 9 times with ethyl acetate. The combined organic layers were concentrated *in vacuo* to afford the corresponding crude acid directly engaged in the next step without further purification.

RMN ^31^P (CDCl_3_, 202 MHz): δ 21.01.

To a solution of the crude acid (211 mg, 0.84 mmol) in dichloromethane (8.40 mL, C = 0.1 M) were successively added ethyl chloroformate (0.093 mL, 0.94 mmol) and pyridine dropwise (0.084 mL, 1.02 mmol) with vigorous stirring. After gas evolution, the mixture was refluxed for 20 min and then cooled down to r.t. The mixture was then washed with an aqueous 0.2 M solution of HCl and the aqueous phase was extracted 3 times with CH_2_Cl_2_. The combined organic layers were dried (MgSO_4_), filtered and concentrated *in vacuo* then purified over silica gel flash chromatography to afford the ethyl phosphinate **Dj** as a pale yellow oil (84 mg, 36 % yield over 2 steps).

RMN ^1^H (CDCl_3_, 500 MHz): δ 7.70 (d, *J* = 439 Hz, 1H), 7.74 (dd, J = 8.5 Hz, 13 Hz, 2H), 7.13-7.06 (m, 2H), 7.06-7.00 (m, 4H), 4.23-4.09 (m, 2H), 1.39 (t, *J* = 7.5 Hz, 3H). ^31^P (CDCl_3_, 202 MHz): δ 23.85. ^19^F (CDCl_3_, 470 MHz): δ -117.9.

*Synthesis of* ***Da***: Compound **Da** was obtained from commercially available phenyl phosphinic acid (1.00 g, 6.93 mmol) according to the same conditions as described above and was isolated as a yellow oil (1.15 g, 98%).

RMN ^1^H (CDCl_3_, 500 MHz): δ 8.12-6.83 (m, 6H), 4.15-3.97 (m, 2H), 1.36-1.19 (m, 3H). RMN ^31^P CPD (CDCl_3_, 202 MHz): δ 24.47.

*Synthesis of* ***Ea****:* In a screw tube, compound **Da** (200 mg, 1.18 mmol), triethylamine (500 µL, 3.6 mmol), vinyl bromide (1.3 mL, 1.3 mmol, 1 M in THF) and palladium dichloro bis(triphenyl phosphine (38.5 mg, 4.7 mol %) were successively introduced. The tube was sealed and heated to 100 °C for 1.5 h. After cooling down to r.t., the mixture as diluted with EtOAc and the solid was filtered off. The organic layer was washed with aqueous HCl (0.5 M, 2 x 5 mL). The aqueous phase was extracted with EtOAc and the combined organic layers were dried (MgSO_4_), filtered and concentrated in vacuum. Flash chromatographic purification of the crude (Cyclohexane/EtOAc 20/80 to 0/100) afforded compound **Ea** as a yellow oil (200 mg, 87% yield).

RMN ^1^H (CDCl_3_, 500 MHz): δ 7.83-7.74 (m, 2H, H_Ar_), 7.58-7.50 (m, 1H, H_Ar_), 7.50-7.42 (m, 2H, H_Ar_), 6.37-6.05 (m, 3H, H_alkene_), 4.15-3.94 (m, 2H, CH_2_), 1.33 (t, *J* = 7 Hz, 3H, CH_3_). RMN ^31^P (CDCl_3_, 202 MHz): δ 29.95.

*Synthesis of compound* ***Fm****:* In a screw tube ethyl phosphinate **Dj** (84 mg, 0.310 mmol) and ethyl vinyl phenyl phosphinate **Ea** (50 mg, 0.25 mmol) were placed in anhydrous THF (1.8 mL, C = 0.14 M) under argon. The *t*BuOMgCl solution (0.280 mL, C = 0.66 M in THF, freshly prepared from distilled *t*BuOH and *i*PrMgCl in THF) was then added and the solution was stirred at room temperature for 72 h. The mixture was then quenched by the addition of a saturated aqueous solution of NH_4_Cl. The aqueous phase was extracted 3 times with CH_2_Cl_2_. The combined organic layers were dried over MgSO_4_, filtered and concentrated *in vacuo*. The crude residue was purified over silica gel flash chromatography to give the product **3** as an oil (28 mg, 23 % yield).

RMN ^1^H (CDCl_3_, 500 MHz): δ 7.85-6.85 (m, 13H), 4.13-3.93 (m, 2H), 3.92-3.70 (m, 2H), 2.30-2.08 (m, 2H), 2.06-1.89 (m, 2H), 1.30-1.21 (m, 6H). RMN ^31^P CPD (CDCl_3_, 202 MHz): δ 42.9 (m). ^19^F (CDCl_3_, 470 MHz): δ -118.0.

*Synthesis of compound* ***G1****:* The bis-phosphinate **Fm** (28 mg, 0.059 mmol) was suspended in concentrated HCl (0.500 mL, 6.00 mmol) and stirred at 80 °C for 72 h. The mixture was cooled at room temperature then MeOH was added and the resulting solid was filtered and successively washed with a minimum quantity of H_2_O followed by diethyl ether. The solid was dried *in vacuo* to afford the diacid **G1** as a white solid (13 mg, 53 % Yield). The diacid (13 mg, 0.031 mmol) was then placed in pure water (3.4 mL, C = 0.01 M) and sodium hydroxide (2.46 mg, 0.061 mmol) was added. The suspension was stirred at r.t. until complete solubilisation. After freeze drying and drying over P_2_O_5_, the disodium salt was obtained as a white solid (14 mg, quantitative yield).

RMN ^1^H (D_2_O, 500 MHz): δ 7.68-7.46 (m, 7H, H_Ar_), 7.32-7.15 (m, 4H, H_Ar_), 7.07 (d, J = 7.5 Hz, 2H, H_Ar_), 1.74 (brs, 4H, 2 CH_2_). RMN ^31^P (Composite pulse decoupling) (D_2_O, 202 MHz): δ 34.39 (m). ^19^F (CDCl_3_, 470 MHz): δ -119.4.

**References**

Garst, M.E. Alkylation of phenyl phosphinic acid. *Synthetic Commun*  **9**, 216-266 (1979)

Harwood, H.J., Grisley, Jr.D.W. The unexpected course of several Arbuzov-Michaelis reactions; an example of the nucleophilicity of the phosphoryl group. *J Am Chem Soc* **82**, 423-426 (1960)

Abramov, V.S., Tarasov, L.A., Fatykhova, F.G. Reactions of sodium salts of phenylphosphonous acid monoesters with some α,ω-dihalo alkanes. I. Synthesis of p,p'-alkylene-p,p'-diphenyldiphosphonates. *Zh Obshch Khim* **38**, 1794-1798 (1968)

Montchamp, J.L., Dumond, Y.R. Synthesis of monosubstituted phosphinic acids: Palladium-catalyzed cross-coupling reactions of anilinium hypophosphite. *J Am Chem Soc* **123**, 510-511 (2001)

Afarinkia, K., Yu, H-w. Hewitt reaction revisited. *Tetrahedron Lett* **44**, 781-783. (2003)

Xu, Y., Li, Z. Palladium-catalysed synthesis of alkyl alkenylmethyl- and alkenylphenylphosphinates. *Synthesis* 240-242 (1986)

Han, L.B., Zhao, C.Q. Stereospecific addition of H-P bond to alkenes: a simple method for the preparation of (RP)-phenylphosphinates. *J Org Chem* **70**, 10121-10123 (2005)

**Supplementary Table 1.** Conditions and yields for the synthesis of compound **c407** (**A2**) and analogs **A1**, **A3**, **A4**

| **Entry** | **n** | **X** | **Base** | **Duration** | **Compound** | **Yield*^a^*** |
| --- | --- | --- | --- | --- | --- | --- |
| 1 | 1 | Br | *n*BuLi | 22 h | **A2** | None |
| 2 | 1 | Cl | *n*BuLi | 22 h | **A2** | 15 % |
| 3 | 1 | Cl | LDA | 42 h | **A2** | 36 % |
| 4 | 1 | Cl | LDA | 66 h | **A2** | 59 % |
| 5 | 0 | Cl | LDA | 66 h | **A1** | 13 % |
| 6 | 2 | Cl | LDA | 66 h | **A3** | 31 % |
| 7 | 3 | Cl | LDA | 66 h | **A4** | 70 % |

*^a^*: Isolated yield after work-up and acidification with HCl 6N.

**Supplementary Table 2.** Conditions and yields for the synthesis of intermediates **Dx** and **Ex**

| **Ar** | **Duration of reaction a (h)** | **Catalyst**  **(mol %)** | **Crude acid (yield %)** | **Compound Dx (yield %)*^*^*** | **Compound Ex (yield %)*^*^*** |
| --- | --- | --- | --- | --- | --- |
| Phenyl | - | - | Commercial | **Da** (98) | **Ea** (87) |
| 4-(Me)-phenyl | 4 | 0.6 | 77 | **Db** (94) | **Eb** (87) |
| 4-(CO_2_Et)-phenyl | 21 | 0.2 | 80 | **Dc** (99) | **Ec** (70) |
| 1-(MeO)-phenyl | 16 | 2.2 | 91 | **Dd** (52) | **Ed** (93) |
| 4-(MeO)-phenyl | 20 | 2.2 | 88 | **De** (63) | **Ee** (87) |
| 4-Cl-phenyl | 4 | 0.6 | 84 | **Df** (71) | **Ef** (83) |
| 4-Br-phenyl | 5 | 0.6 | 49 | **Dg** (63) | **Eg** (37) |
| 1-F-phenyl | 21 | 2 | 40 | **Dh** (78) | **Eh** (66) |
| thienyl | 8*^**^* | 0.6 | 61 | **Di** (65) | **Ei** (80) |

*^*^*Isolated yield after flash chromatographic purification.

*^**^* The stirring was continued at r.t. for 48 h.

**Supplementary Table 3.** Yields for the synthesis of protected **c407** analogs **Fx** and **c-407** analogs **C1-C4**, **C8-C12**

| **Reagents** | | **Products** (yield) | | | **Ar^1^** | **Ar^2^** |
| --- | --- | --- | --- | --- | --- | --- |
| **Dx** | **Ex** | **Fx**  (yield %) | **Cx**  (yield %) | **Na^+^ salts** (yield %) |  |  |
| **Da** | **Ea** | **Fa** (81) | **c407** (91) | 100 | Phenyl | |
| **Db** | **Eb** | **Fb** (70) | **C1** (37) | 96 | 4-(Me)-phenyl | |
| **Dc** | **Ec** | **Fc** (56) | **C2** (53) | 98 | 4-(CO_2_H)-phenyl | |
| **Dd** | **Ed** | **Fd** (71) | **C3** (67) | 100 | 1-(MeO)-phenyl | |
| **De** | **Ee** | **Fe** (85) | **C4** (65) | 100 | 4-(MeO)-phenyl | |
| **Df** | **Ef** | **Ff** (90) | **C6** (94) | 97 | 4-Cl-phenyl | |
| **Dg** | **Eg** | **Fg** (91) | **C7** (88) | 94 | 4-Br-phenyl | |
| **Dh** | **Eh** | **Fh** (90) | **C8** (75) | 100 | 1-F-phenyl | |
| **De** | **Ea** | **Fi** (90) | **C9** (67) | 100 | phenyl | 4-(MeO)-phenyl |
| **De** | **Ef** | **Fj** (88) | **C10** (77) | 98 | 4-Cl-phenyl | 4-(MeO)-phenyl |
| **Di** | **Ea** | **Fk** (89) | **C11** (93) | 100 | phenyl | thienyl |
| **Di** | **Ei** | **Fl** (83) | **C12** (77) | 94 | thienyl | |

**Supplementary Figure Legends**

**Supplementary Figure 1. Correction of CFTR activity in primary respiratory cells incubated with c407, its extended derivative G1 and VX-809.**

Mean(SEM) of the Isc response to Forskolin (10 μM)/IBMX (100 μM) + VX-770 (10 μM) after treatment of human nasal or bronchial cells of F508del homozygous patient after incubation with c407 10µM (n=7) or 50 µM (n=11), G1 50µM (n=14), VX-809 3µM (n=32) for 48 h, and their respective diluent Ringer (n=12) or DMSO (n=32). Post Hoc Wilcoxon statistics are shown.

**Supplementary Figure 2. Comparison of the model A (according to ^19^) and cryo-EM B (pdb 6MSM) (according to ^20^) 3D structures of the wild-type human CFTR**

The similarities of the NBD1 (light blue) and ICL4 (red) interfaces are shown. The C-alpha positions of the amino acids discussed in this work are similar, with variability observed in the positions of the side chains.

**Supplementary Figure 3. The c407 binding site in isolated NBD1 and in the MSD:NBD assembly.**

Topologies of the c407 binding sites are shown on the left-hand side, whereas contact maps, calculated over MD simulations, are shown on the right-hand side. **A)** The c407 binding site in the context of the isolated F508del NBD1 (extracted from the whole MSDs:NBDs architecture); **B)** The c407 binding site in the context of the F508del MSDs:NBDs assembly.

**Supplementary Figure 4. Impact of F1068A on CFTR maturation**

1. Immunoblot of HEK-293 cells transiently expressing WT, F508del-, F1068A-WT CFTR. and F1068A-F508del CFTR. Arrows indicate CFTR immature core glycosylated band B and complex glycosylated mature band C. CFTR was immunoblotted with monoclonal CFTR antibody 660, at 1:1000. Representative experiments of n=4.

B) Summary of CFTR expression quantification. CFTR expression is evaluated by the ratio C/B+C. The ratio of C/B+C is significantly higher in the HeLa cells expressing WT or F1068A-WT-CFTR *versus* the cells expressing F508del or F508del-F1068A (n=4) (p=0.01 for both).

**Supplementary Figure 5. Retrosynthetic analysis and synthesis of compound analogs A and B.**

A) Retrosynthetic analysis towards analogues of c407 with various chain lengths and rigidity. B) Synthesis of compound c407 (**A2**) and analogs (**A1**, **A3**, **A4**) with various chain lengths.

C) Synthesis of analog **B1** (modification of the chain rigidity).

**Supplementary Figure 6. Retrosynthetic analysis and synthesis of compound analogs C**

A) Retrosynthetic analysis towards c407 analogs with various aromatic or heteroaromatic substituents

B) Reaction conditions for the synthesis of Dx and Ex: a) Pd(PPh_3_)_4_ (0.2-2 mol %), NEt_3_ (3 equiv.), MeCN reflux. b) Ethyl chloroformate (1.1 equiv.), pyridine (1.1 equiv.), DCM reflux (40 min). c) Vinyl bromide (1.2 equiv.), Et_3_N (3 equiv.), Pd(PPh_3_)_2_Cl_2_ (5 mol %), 100 °C, 2 h.

C) Reaction conditions for the synthesis of symmetrical and asymmetrical analogs of compound c407: **C1-C4, C6-C12:** a) *t*BuOMgCl 20 mol%), THF, rt. b) HCl conc., 100 °C. c) NaOH, H_2_O, rt, 10 min.

**Supplementary Figure 7. Synthetic scheme for the synthesis of the extended compound G1.**
A) Path A: Synthesis of the intermediate aryl phosphinic ethyl ester **Dj.**

B) Path B: Synthesis of the intermediate phenyl vinyl phosphinate **Ea**.

C) Path C: Synthesis of the targeted extended c407 analog **G1** from **Dj** and **Ea**

**Supplementary Figure 8. Impact of VX-809, c407 and the 16 synthesized derivatives on F508del-CFTR maturation.**

A) Immunoblot of HeLa cells stably expressing WT and F508del-CFTR. Cells were incubated for 48 hours with VX-809 at 3 µM, c407 or its derivatives at 10µM. Arrows indicate CFTR immature core glycosylated band B and complex glycosylated mature band C. CFTR was immunoblotted with monoclonal CFTR antibody 660, at 1:1000. Representative experiments of n=4.

B) Summary of CFTR expression quantification. CFTR expression is evaluated by the ratio C/B+C. The ratio of C/B+C is significantly higher in F508del-HeLa cells incubated with VX-809 µM at 3 µM (p=0.007) or c407 10µM (p=0.015) versus F508del-HeLa cells respectively incubated with control water (untreated) (n=8) or DMSO (n=16). The other compounds did not provide a significant increase in the C/B+C ratio (n=4). Post hoc wilcoxon statistics are shown.

**Supplementary Figure 9. C407 and VX-809 combination in HeLa cells assessed by whole cell patch-clamp experiments.**

Mean(SEM) CFTR current amplitudes were recorded at -60 mV and normalized to cell capacitance in HeLa cells stably expressing CFTR. Current densities were normalized to cell capacitance (I _CFTR_/C) and calculated as the differences between current values in the presence of CPT-cAMP 400 µM/IBMX 100 µM minus current values after inhibition with CFTRinh-172 5 µM. Results are shown for WT cells (black dotted; n=12); F508del untreated cells (empty; n=5), F508del cells treated for 48 h by: c407 10 µM (black; n=9), VX-809 1 µM (loose hatching; n=8); VX-809 3 µM (tight hatching; n=5) ; c407 10 µM + VX-809 1µM (loose cross-hatching; n=8); c407 10 µM + VX-809 3 µM (tight cross-hatching; n=3). Post hoc Wilcoxon statistics are shown.

Supplementary Figure 1


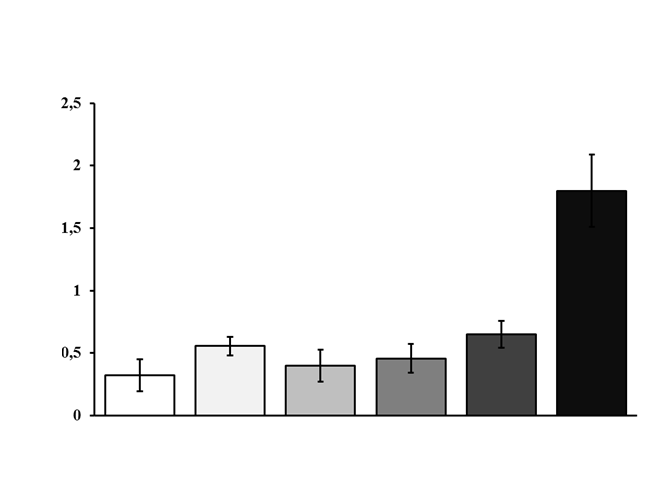


p<0.0001

p=0.04

p=0.005

p=0.001

p=0.02

p=0.02

Isc Forskolin/IBMX response (µA/cm^2^)

2.5

2

1.5

1

0.5

0

Ringer

DMSO

c407 10 µM

c407 50 µM

G1 50 µM

VX-809 3 µM

Supplementary Figure 2


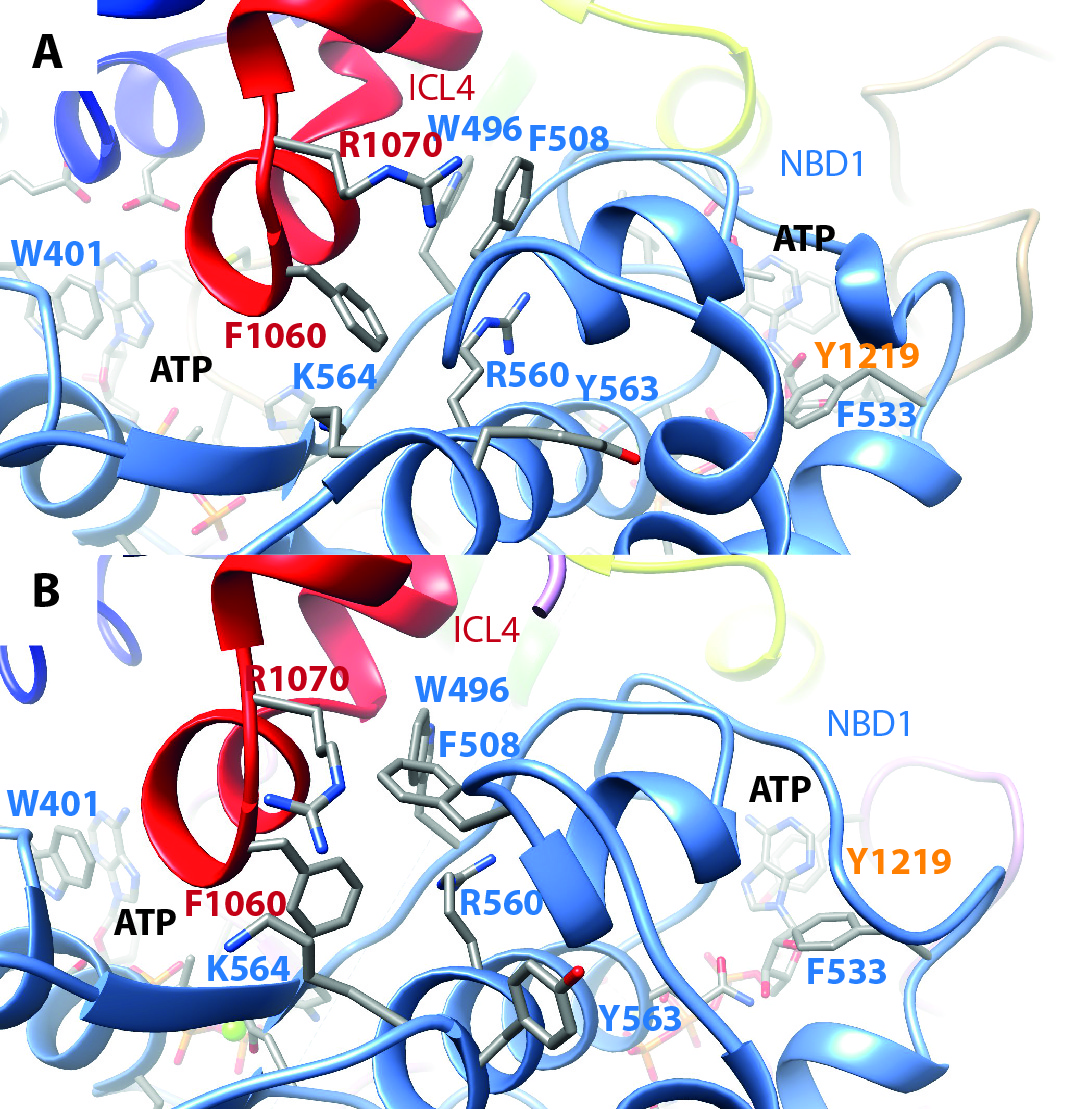


Supplementary Figure 3


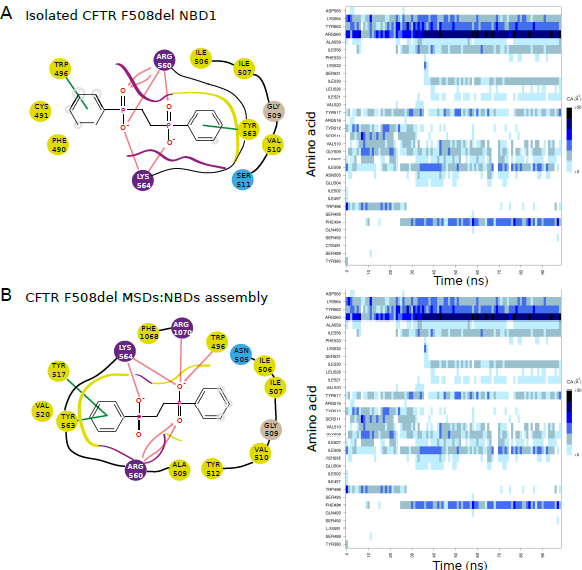


Supplementary Figure 4


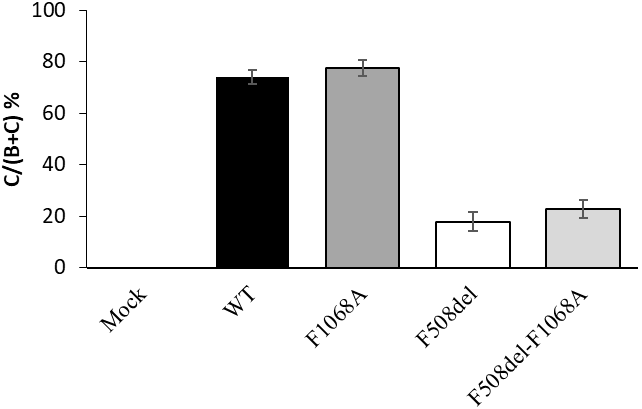

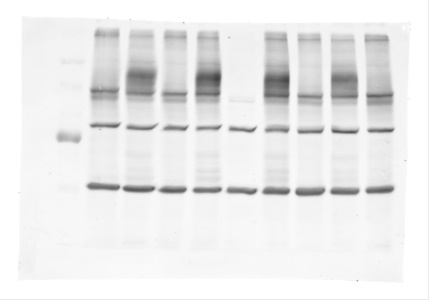


WT

Mock

F508del

F1068A

F508del-F1068A

Tubulin

250 kDa

150 kDa

50 kDa


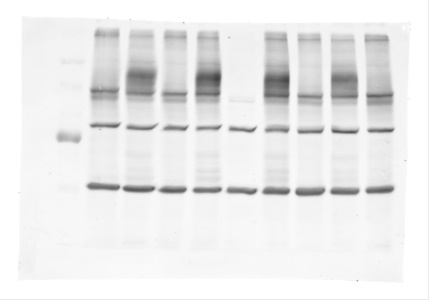


Band C

Band B

*

*

Supplementary Figure 5

**B**

**A**

**C**

Supplementary Figure 6

**A**

**B**

**C**

Supplementary Figure 7

Supplementary Figure 8

CFTR

Tubulin


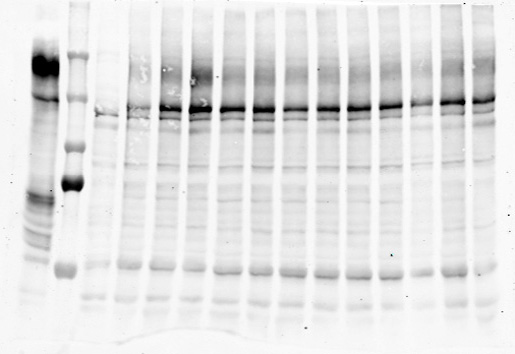

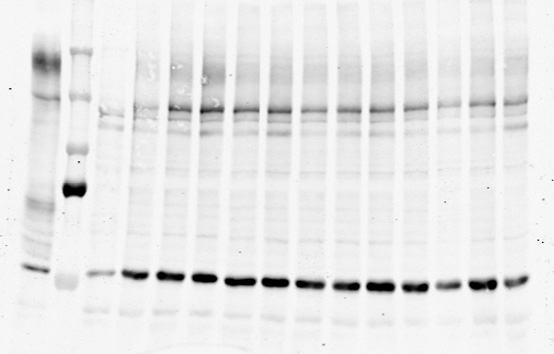


C5

standard

WT

Mock

c-407

C4

C3

C8

C11

C9

C10

B1

VX-809

DMSO

Untreated

F508del

standard

WT

Mock

c-407

C1

C2

C12

C6

C7

A3

A4

Untreated

VX-809

DMSO

F508del

A1

250 kDa

150 kDa

100 kDa

75 kDa

50 kDa

Band C

Band B


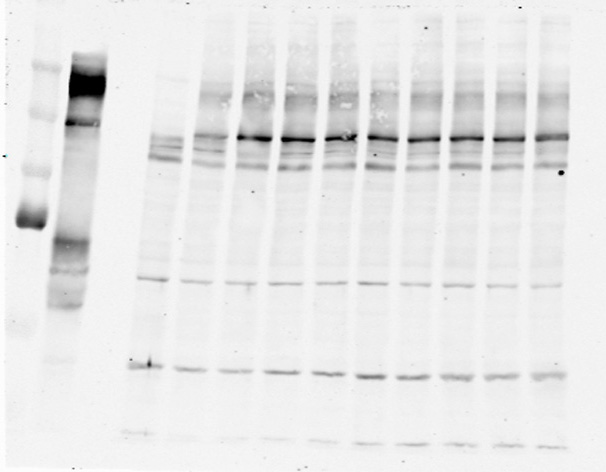

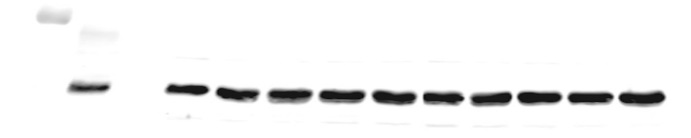

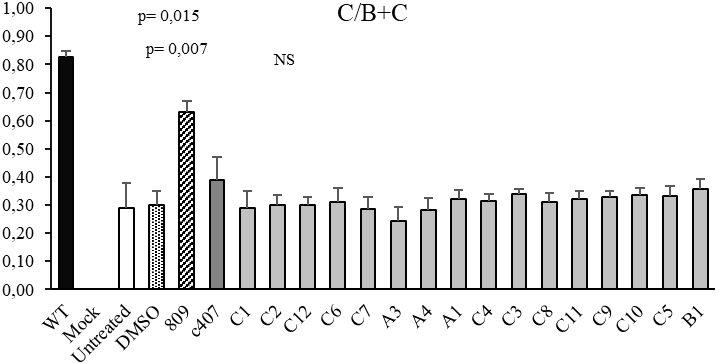


n = 4

n = 8

n = 16

n = 16

*** p<0.001

** p<0.01

* p<0.05

*** p<0.001

* p<0.05

** p<0.01

Supplementary Figure 9


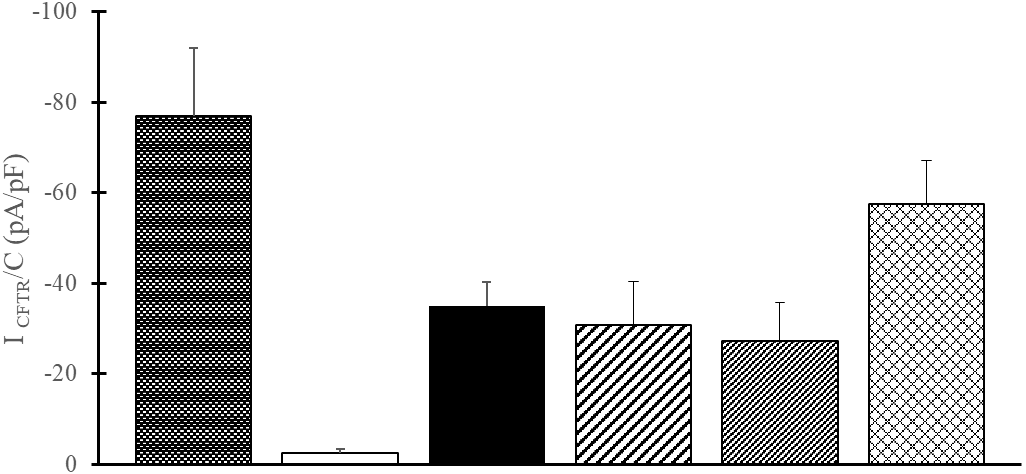


WT

F508del

c407 10µM

VX-809 1µM

VX-809 1µM + c407 10µM

VX-809 3µM

VX-809 3µM + c407 10µM

*

**

*

*

***

*

*

*
